# Cardiopulmonary Exercise Performance of Children Born Non-Extremely Preterm

**DOI:** 10.3390/children11020198

**Published:** 2024-02-04

**Authors:** Sotirios Fouzas, Aikaterini Nourloglou, Aggeliki Vervenioti, Ageliki Karatza, Michael B. Anthracopoulos, Gabriel Dimitriou

**Affiliations:** Department of Pediatrics, University of Patras School of Medicine, 26504 Patras, Greece; nourlogla@ac.upatras.gr (A.N.); verveniotia@upatras.gr (A.V.); karatza@upatras.gr (A.K.); manthra@otenet.gr (M.B.A.); gdim@upatras.gr (G.D.)

**Keywords:** cardiopulmonary exercise testing, exercise performance, exercise tolerance, prematurity, preterm children, children

## Abstract

Data on exercise tolerance of children born non-extremely preterm are sparse. We aimed to explore the cardiopulmonary exercise testing (CPET) characteristics in this population. We studied 63 children (age 7–12 years) born at 29^0/7^–36^6/7^ weeks of gestation (34 were late preterm, 29 were preterm) and 63 age-matched, term-born controls. All performed spirometry and CPET (cycle ergometry). There were no differences in activity levels and spirometric parameters between the group of preterm-born children and controls. A peak oxygen uptake (VO_2_peak) of <80% was noted in 25.4% of the term-born and 49.2% of preterm-born children (*p* = 0.001). Term-born participants presented similar VO_2_peak to late-preterm children but higher than those born at <34^0/7^ weeks of gestation (*p* = 0.002). Ventilatory limitation was noted in 4.8% of term and 7.9% of preterm participants, while only one preterm child presented cardiovascular limitation. Children born before 34 weeks of gestation had higher respiratory rates and smaller tidal volumes at maximum exercise, as well as lower oxygen uptake for the level of generated work. We conclude that school-age children born at 29–34 weeks of gestation may present decreased exercise performance attributed to an altered ventilatory response to exercise and impaired O_2_ utilization by their skeletal muscles rather than other cardiopulmonary limiting factors.

## 1. Introduction

Prematurity, defined as delivery before completing the 37 weeks of gestation, represents a global health concern [1]. Besides the significant postnatal morbidity and mortality, preterm-born infants may face lifelong medical challenges, including neuro-developmental, pulmonary, cardiovascular, and metabolic impairment [2,3]. Even though advancements in perinatal care have ameliorated these short- and long-term consequences [1,2], prematurity may still be associated with pulmonary and cardiovascular alterations [4] that deter their daily activities, including tolerance to exercise [2,3].

To date, most studies using cardiopulmonary exercise testing (CPET) to assess the exercise performance of ex-preterm children have focused on those born at <28 [5,6,7,8,9] or <32 [10,11,12,13,14] weeks of gestation. Thus, a significant body of evidence suggests that these children may attain lower maximum workload (Wmax) [5,6,7], lower peak VO_2_ uptake (VO_2_peak) [13,15,16], and abnormal ventilatory responses to exercise [5,9,13] than their term-born counterparts; these alterations are more prominent in individuals diagnosed with bronchopulmonary dysplasia (BPD) [9,13,15]. Data on the exercise performance of children born later than the lower extremes of prematurity are sparse and conflicting [16,17,18]; one study has shown significant differences in VO_2_peak and other CPET parameters compared to term-born controls [18], while others have failed to confirm the above results [16,17]. Nevertheless, objective data on the exercise capacity of the more “mature” preterm children are important because they represent almost 90% of the rising ex-preterm population [19,20], and any impairment in exercise tolerance may lead to physical inactivity with long-term consequences for their health and well-being [21].

The aim of the present study was to explore the CPET characteristics of school-age children born non-extremely preterm in comparison to age-matched, term-born controls. We hypothesized that these children may present subtle and underrecognized CPET abnormalities that might significantly affect their exercise tolerance and performance.

## 2. Materials and Methods

### 2.1. Study Design

This was a cross-sectional study of term- and preterm-born children aged 7–12 years, who were matched for age and sex. This study was conducted at the CPET laboratory of the Pediatric Respiratory Unit of the University Hospital of Patras, Greece, between June 2017 and December 2022. Participants were recruited from the CPET laboratory (children referred to estimate their exercise performance) and from the outpatient pediatric clinics of our hospital. All should have had no asthma diagnosis or prescription of anti-asthmatic medication within the last two years, normal spirometry at enrolment, and normal electrocardiogram and echocardiographic examinations. Exclusion criteria were (1) gestational age (GA) < 28 complete weeks; (2) history of BPD (oxygen requirement of >28 days); (3) asthma/wheezing within the last two years; (4) cardiovascular disease; (5) significant disabilities (neurological, muscular, neurodevelopmental); (6) lower respiratory infection (i.e., bronchitis or pneumonia) in the last month; and (7) non-specific respiratory symptoms in the week before the visit.

This study was approved by the Ethics Committee of the University Hospital of Patras (Act no. 156/03.02.2017). The parents of the children were informed in detail about the purposes and requirements of the study, and parental written consent and participants’ verbal assent were obtained before enrollment.

### 2.2. Demographics and History

Demographics were collected on the day of the study visit. Height and weight were measured, and body mass index (BMI) was calculated and assessed according to the International Obesity Task Force guidelines [22]. Participants’ health booklets were carefully reviewed by one of the investigators to obtain all available information regarding (1) gestational age (GA) at birth and perinatal events such as admission to the Neonatal Intensive Care Unit (NICU), the need for mechanical ventilation (MV), the need and duration of O_2_ support, and (2) a history of wheezing/asthma or relevant medication. When the booklet recordings were incomplete, the parents were interviewed explicitly about the above topics; if inconsistencies persisted, the child was not included in the study.

### 2.3. Physical Activity Status

The parents of the participants were asked to complete the short form of the International Physical Activity Questionnaire—Greek version (IPAQ-SF-GR) [23] to estimate the frequency, duration, and intensity of their children’s physical activity. Participants with at least 60 min of moderate-to-vigorous daily physical activity were considered “active”; otherwise, they were classified as “not active” [21].

### 2.4. Spirometry

Spirometry was performed before CPET with a Micro 5000 spirometer (Medisoft, Sorinnes, Belgium) according to the guidelines [24]. The forced expiratory volume at 1 s (FEV1), forced vital capacity (FVC), FEV1/FVC ratio, and forced expiratory flow between 25 and 75% of FVC (FEF25–75) were recorded and assessed according to Global Lung Initiative norms [25]. Spirometry was repeated at 5, 10, 15, and 20 min after CPET, and a decrease in FEV1 of ≥15% was defined as exercise-induced bronchoconstriction (EIB).

### 2.5. Cardiopulmonary Exercise Testing

CPET was performed in an air-conditioned room (temperature 20–23 °C, relative humidity 50–60%) using an Ultima CPX device (MGC Diagnostics, Saint Paul, MN, USA) with a cycle ergometer (eBike, GE Healthcare, Chicago, IL, USA). After an accommodation period of 5 min followed by 3 min of free pedaling, the workload began to increase automatically by a constant rate (ramp) of 15 or 20 watts/min according to the subject’s height (<150 or ≥150 cm, respectively), while the child was instructed to maintain a stable pedaling speed of 60–65 rpm up to physical exhaustion [26]. Continuous electrocardiographic, blood pressure, and oxygen saturation (SpO_2_) monitoring was also applied. The trial was terminated when the subject could not maintain the pedaling pace despite encouragement or when adverse events such as chest pain, excessive dyspnea, electrocardiographic alterations, or significant desaturation (SpO_2_ < 92%) occurred [27].

The following variables were recorded: work (W, in watts), oxygen uptake (VO_2_, in mL/min), carbon dioxide output (VCO_2_, in mL/min), respiratory exchange ratio (RER), heart rate (HR, in beats per min), respiratory rate (RR, in breaths per min), tidal volume (VT, in L), and minute ventilation (VE, in L/min). All variables were obtained breath by breath, averaged over eight consecutive breaths, and their maximum (“peak” or “max”) values were recorded. The anaerobic threshold (AT) was determined by the V-slope method, and the VO_2_AT and VCO_2_AT were computed. The ratios VO_2_peak/Wmax and VO_2_peak/HRmax (oxygen pulse) were calculated, and the breathing reserve was defined as 100 − (VEmax × 100)/(30 × FEV1) [28]. The ventilation efficiency slope (VE/VCO_2_ slope) and the oxygen uptake efficiency slope (OUES, calculated as VO_2_/logVE) were also obtained. All CPET variables, except VO_2_peak/Wmax and OUES, were expressed as % predicted values according to recently published normative data [29]. The level of breathing discomfort at maximum exercise was assessed by the Borg scale.

A CPET was considered maximal if (1) signs of maximum effort (sweating, fatigue) were present, (2) the HR was ≥85% of predicted, and (3) the RER was >1.10. Ventilatory limitation was defined as breathing reserve < 15% in a subject with VO_2_peak < 80% of that predicted. Cardiovascular limitation was defined as an early plateau or drop of oxygen pulse before peak exercise in a child with VO_2_peak < 80% of predicted. Participants with a VO_2_peak < 80% but without ventilatory or cardiovascular limitation were classified as having “peripheral” limitation, including physical deconditioning [30,31].

### 2.6. Statistics

Participants were assigned to two groups: (1) term group, including children born at ≥37^0/7^ weeks of gestation (i.e., at term); (2) combined preterm group, consisting of participants born before 37^0/7^ weeks of gestation. The latter was further divided into (a) a late-preterm group, including children delivered at a GA of 34^0/7^–36^6/7^ weeks, and (b) a preterm group, consisting of children born at <34^0/7^ weeks of gestation.

Continuous variables are presented as mean ± SD and with range. After normality testing with the Shapiro–Wilk and Kolmogorov–Smirnov tests, comparisons between groups were performed with the Mann–Whitney U test (term vs. combined preterm group) or the Kruskal–Wallis test with Dunn’s multiple comparison analysis (term vs. late-preterm vs. preterm group). The chi-square or Fisher’s exact tests were used to compare categorical variables. Statistical analyses were performed using IBM SPSS version 28 (IBM Corp., Armonk, NY, USA).

## 3. Results

Overall, 140 term- and preterm-born children matched for age and sex fulfilled the inclusion criteria and were enrolled in the study. Three children (two born preterm) had incomplete or inconsistent perinatal data, while four participants (two born preterm) performed a submaximal test; all were excluded from the analysis together with their matched counterparts. Therefore, the final study population consisted of 63 term- and 63 preterm-born children. Of the latter, 34 were assigned to the late-preterm group and 29 to the preterm group. Their general characteristics are presented in Table 1.

Children of the combined preterm group had a higher rate of NICU admission (*p* < 0.001) and need for MV (*p* < 0.001) or O_2_ support (*p* < 0.001) compared to those of the term group (Table 1). The prevalence of wheezing/asthma was similar between the two groups (*p* = 0.374), as was the percentage of systematic participation in sports (*p* = 0.571). According to IPAQ, 79.4% of the children in the term group and 69.8% of those in the combined preterm group were classified as “active” (*p* = 0.302) (Table 1).

There were no differences in spirometric parameters between the study groups (Table 2).

**Figure 1:**
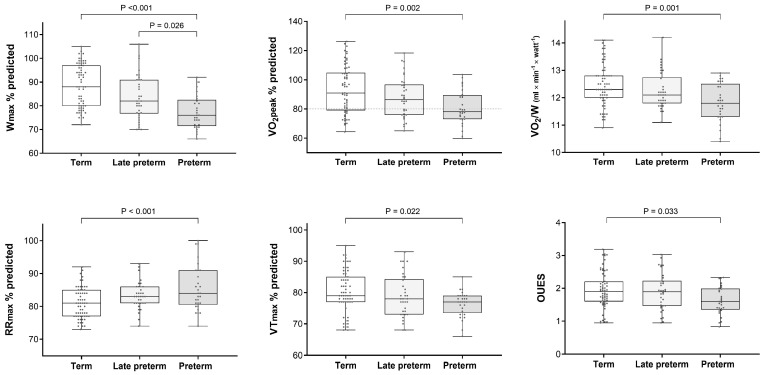
Comparison of CPET parameters between term, late-preterm (GA 34^0/7^–36^6/7^ weeks), and preterm (GA 29^0/7^–33^6/7^ weeks) groups. Box and Whisker plots (mean, interquartile range, and range) including the raw data (dots). Statistical significance was assessed by Dunn’s multiple comparison test. Only *p*-values < 0.05 are presented.

Cardiopulmonary parameters before CPET (Table 3) were no different between the study groups. CPET results are presented in Table 4 and Figure 1. Term-born participants had higher Wmax, VO_2_peak, VO_2_/W, and VTmax but lower RRmax than their counterparts in the combined preterm group. Term- and late-preterm-born children had higher Wmax and VO_2_/W than those born at <34^0/7^ weeks of gestation (Figure 1). The VO_2_peak and the VTmax were higher, and the RRmax was lower in the term group only in comparison to the preterm group with GA < 34^0/7^ weeks (Figure 1). The OUES was also higher in term-born participants than those born at <34^0/7^ weeks of gestation (Figure 1).

**Figure 2:**
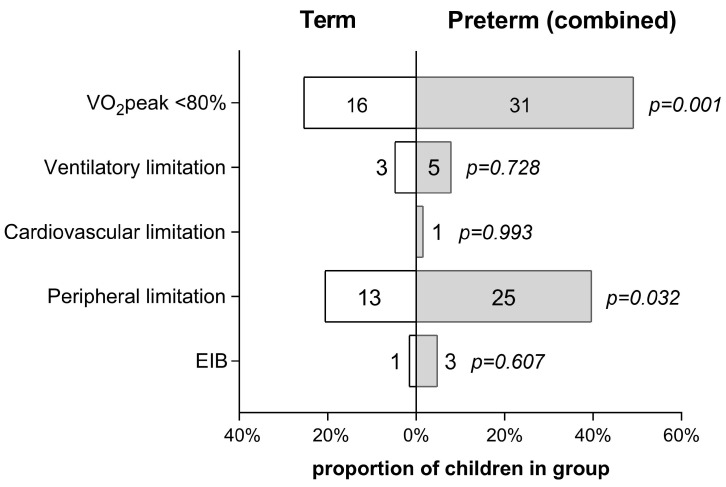
Physical activity status and CPET-derived characteristics of the term and combined preterm group. The number of cases is presented within or alongside the bars. EIB: exercise-induced bronchoconstriction.

A total of 16 children in the term group and 31 in the combined preterm group (25.4 and 49.2%, respectively; *p* = 0.001) had a VO_2_peak < 80% of the predicted value (Figure 2). Ventilatory limitation was noted in three participants (4.8%) of the term group and five (7.9%) of the combined preterm group, while one preterm child (1.6%) presented cardiovascular limitation. Thus, peripheral limitation emerged as the leading cause of reduced cardiopulmonary exercise performance in 13 (20.6%) children in the term group and 25 (39.7%) in the combined preterm group (*p* = 0.032). EIB was noted in one child (1.6%) of the term group and three (4.8%) of the combined preterm group (Figure 2).

## 4. Discussion

In this case–control study, we assessed the exercise capacity of school-age children born non-extremely preterm (i.e., between 29^0/7^ and 36^6/7^ weeks of gestation) in comparison to age-matched, term-born controls. We found that the preterm participants attained lower Wmax, VO_2_peak, VO_2_/W, and OUES and adopted a more rapid and shallow breathing pattern (i.e., higher RR—lower VT) at maximum exercise compared to controls. The above differences were mainly observed between children born before 34 weeks of gestation and their term-born counterparts, while those born late preterm (i.e., at a GA 34^0/7^–36^6/7^ weeks) presented CPET outcomes similar to controls. Abnormal VO_2_peak values (i.e., <80% of the predicted value) were also more prevalent in preterm-born children.

### 4.1. Effect of Cardiopulmonary Limiting Factors

The reduced exercise capacity of our preterm group could not be attributed to cardiopulmonary limiting factors (Figure 2), such as cardiovascular or respiratory disease or expiratory flow limitation (EFL). All participants had normal baseline electrocardiographic and echocardiographic examination, and there were no differences in HRmax, O_2_ pulse, and SpO_2_peak between groups (Table 4); therefore, the contribution of cardiac or pulmonary vascular factors is highly unlikely. Baseline spirometric indices, breathing reserve, VE/VCO_2_ slope, and the prevalence of EIB were also similar between the study groups (Table 4, Figure 2), suggesting that ventilatory limitation could also not be responsible for the observed differences in exercise capacity. Previous CPET-based studies have shown that EFL is common and may be a major limiting factor in children who were born extremely preterm and were diagnosed with BPD [9,13]. However, the prevalence of EFL did not differ between their non-BPD counterparts and term-born controls, suggesting that EFL is related to the degree of pulmonary injury rather than prematurity per se [9,13]. Thus, although we did not include flow-volume loop analysis to specifically assess EFL [9,13], its effect—if any—was most likely minor.

### 4.2. Ventilatory Response to Exercise

A key finding of our study was the more rapid and shallow breathing pattern of preterm-born children at maximum exercise, as reflected by their higher RRmax and lower VTmax (Table 4, Figure 1). Of note, there were no differences in RR and VT between the study groups before the CPET (Table 3); therefore, this unusual breathing type occurred as a response to exercise. A similar respiration pattern has been observed in extremely preterm children performing CPET [5,7,9], but the underlying mechanism remains unclear. It has been suggested that prematurity is associated with structural and functional airway injury that, under demanding ventilatory conditions, not only increases the resistance to airflow but also causes dynamic hyperinflation and additional elastic loading [11]. Thus, a more rapid and shallow breathing strategy may be necessary to overcome the combined resistive and elastic loads during intense exercise [5]. However, the fact that VEmax, breathing reserve, and PETCO_2_max were comparable between the three study groups (Table 3) suggests that this adaptive mechanism efficiently met the increased ventilatory demands of preterm children without altering the performance of the respiratory pump; therefore, its contribution to exercise limitation is questionable. Alternatively, a more rapid and shallow breathing pattern during intense exercise has been attributed to the fatigability of the respiratory muscles [7]. It has been shown that the inspiratory muscles of children who were born with very low birth weight (<1500 g) present decreased strength at rest (i.e., generate lower maximum inspiratory pressure) and perform less efficiently (i.e., have a higher tension–time index) for the same level of exercise [7]; this “dysfunction” related directly to the lower lean body mass of those children and emerged as the principal determinant of their rapid breathing pattern at maximum exercise [7]. In exercising healthy individuals, respiratory muscle fatigue may induce metaboreflexes that cause peripheral vasoconstriction, promote locomotor muscle fatigue, and eventually lead to exercise limitation [32]. Therefore, we speculate that a lower threshold of the inspiratory muscles to fatigue might explain both the unusual ventilatory response and the reduced exercise capacity of the preterm-born children of our cohort.

### 4.3. Peripheral O_2_ Utilization

Another intriguing finding was the decreased O_2_ uptake for the generated work (i.e., VO_2_/W) in children born before 34 weeks of gestation (Table 4, Figure 1). Although no differences in VO_2_/W have been previously reported between preterm children and controls [7,11], it has been shown that the lower lean body mass of extremely preterm children is directly related to a lower VO_2_peak, suggesting that the metabolic activity of their muscles may be decreased [11]. Indeed, a lower energy cost per work rate indicates an inefficient O_2_ utilization by the skeletal muscles during exercise, which could also contribute to exercise intolerance [33]. The lower OUES in the same group (Table 4, Figure 1) also suggests an impairment in peripheral oxygen extraction, given the absence of cardiopulmonary disease [34]. Whether the above findings relate to structural and functional abnormalities of the skeletal muscles and how these relate to prematurity remains to be determined.

### 4.4. Mechanisms of Exercise Limitation

Taken together, our results suggest that preterm children, especially those born before 34 weeks of gestation, may experience exercise limitation by two less-recognized mechanisms: an altered ventilatory response to exercise that might be attributed to the fatigability of the respiratory muscles and an impairment in O_2_ utilization by the skeletal muscles. Both are included in the umbrella term “peripheral limitation” (i.e., abnormal VO_2_peak in the absence of cardiovascular or ventilatory limitation) [11] that emerged as the leading cause of reduced exercise performance in our study (Figure 2). Although peripheral limitation refers classically to physical deconditioning [30,31] (e.g., due to sedentary behaviors and physical inactivity), we feel that the preterm children of our study were not less fit than their term-born counterparts: they reported similar levels of daily physical activity and comparable or even higher rates of participation in organized sports (Table 1), and they presented no differences in VO_2_AT—a standard CPET index for assessing aerobic fitness [30,35] (Table 4). Nevertheless, it is difficult to determine the degree of contribution of each of the herein-proposed mechanisms or whether they act independently. Future, better-designed, CPET-based studies on respiratory and locomotor muscle strength and performance should further explore the above hypothesis.

### 4.5. Limitations and Strengths

Our study has limitations. First, we have no conclusive data on our participants’ habitual physical activity levels. To keep the study protocol as simple as possible, we collected the relevant information through parental reports using the IPAQ and classified the children as active or inactive. Thus, although there were no significant differences in aerobic fitness between the study groups (see above), we cannot exclude the possibility that some children were more trained than others or that the type of habitual exercise (e.g., bicycling) favored their performance on cycle ergometry. More objective activity data (e.g., physical activity questionnaires in combination with activity tracking devices) would enable us to determine the level of daily physical activity more precisely, explore possible associations with various CPET parameters, and assess whether specific activity patterns would be more suitable for preterm-born children. Second, since we have excluded children with cardiopulmonary disorders and significant disabilities (neurological, muscular, neurodevelopmental, etc.), our results are likely biased towards “healthier” ex-preterm children and cannot be generalized. Third, our study did not include measurements of lung volumes, lung diffusing capacity, blood lactate levels, arterial blood gases, and body composition, which would permit us to explore our hypotheses further. Determining arterial blood gases, in particular, would enable us to estimate the dead space ventilation and explore to what extent its changes may have influenced the VO_2_ in preterm-born children. Nevertheless, the similarities in VE/VCO_2_ slopes between the study groups suggest no significant differences in dead space ventilation; therefore, its effect on the VO_2_ levels in our cohort should be considered minimal. Finally, our population’s relatively wide age range (i.e., 7–12 years) does not permit us to account for the confounding effects of physiologic growth on the CPET outcomes. However, using % predictive [29] instead of raw CPET values should have minimized such influences.

This study is the first to exclusively assess the CPET characteristics of children born non-extremely preterm (i.e., at a GA > 29^0/7^ weeks) in the post-surfactant era. Data on their exercise performance is vital because they represent almost 90% of the ex-preterm population [19,20], and any deficits in exercise tolerance may have significant consequences for their health and well-being [21]. In this regard, our findings may help healthcare and sport-related professionals further understand the functional constraints of preterm infants to exercise, design appropriate physical conditioning programs, and encourage this potentially vulnerable population to promote aerobic fitness.

## 5. Conclusions

School-age children who were born between 29 and 34 weeks of gestation present decreased exercise performance compared to their late-preterm- and term-born counterparts. However, the lower VO_2_peak in this population cannot be attributed to common cardiopulmonary limiting factors. Instead, these children present an altered ventilatory response to exercise (i.e., adopt a more rapid and shallow breathing pattern) and an impairment in O_2_ utilization by their skeletal muscles. These limiting factors may significantly affect the tolerance of preterm-born children to intense or sustained exercise and, thus, warrant further research.

## Figures and Tables

**Table 1:** General characteristics of the study groups.

	Term	Preterm (Combined)	Late Preterm (GA 34^0/7^–36^6/7^ Weeks)	Preterm (GA 29^0/7^–33^6/7^ Weeks)
*n*	63	63	34	29
Male sex, *n* (%)	40 (63.5)	40 (63.5)	21 (61.8)	19 (65.5)
Age, years	9.7 ± 2.1 (7–12)	9.7 ± 2.1 (7–12)	9.9 ± 1.9 (7–12)	9.3 ± 2.5 (7–11)
Height, cm	144.1 ± 8.0 (130–165)	141.9 ± 7.8 (129–163)	143 ± 7.3 (127–162)	140.6 ± 8 (123–155)
Weight, kg	48.5 ± 11.9 (32.5–79)	44.0 ± 10.1 (31–75)	47.6 ± 10 (33–75)	39.8 ± 12 (31–65.4)
BMI, kg/m^2^	24.7 ± 4.4 (16.8–34)	22.1 ± 3.5 (15.6–29.5)	23.6 ± 3.8 (16.3–29.5)	20.3 ± 3.3 (15.6–28)
Overweight, *n* (%)	18 (28.6)	11 (17.4)	6 (17.6)	5 (17.2)
Obese, *n* (%)	7 (11.1)	6 (9.5)	4 (11.8)	2 (6.9)
GA, weeks	38.4 ± 1.1 (37–41)	35.1 ± 2.2 (29–36,9)	35.9 ± 1 (34–36.9)	33 ± 1.2 (29–33.9)
NICU admission, *n* (%)	2 (3.2)	40 (63.5)	11 (32.4)	29 (100)
Need of MV, *n* (%)	0 (0)	14 (22.2)	5 (14.7)	9 (31)
Need of O_2_ support, *n* (%) ^a^	0 (0)	30 (47.6)	10 (29.4)	20 (69)
Days of O_2_ support, *n* (%)	0 (0)	4 ± 5.8 (0.5–18)	3.2 ± 4 (0.5–11)	4.7 ± 6.2 (0.5–18)
History of asthma, *n* (%) ^b^	10 (15.9)	15 (23.8)	7 (20.6)	8 (27.6)
Sports activities, *n* (%) ^c^	39 (61.9)	43 (68.3)	20 (58.8)	23 (79.3)
IPAQ “active”, *n* (%)	50 (79.4)	44 (69.8)	24 (70.5)	20 (68.9)

BMI: body mass index, GA: gestational age, NICU: neonatal intensive care unit, MV: mechanical ventilation, IPAQ: International Physical Activity Questionnaire. Data presented as mean ± SD (range) or number of cases (%). ^a^ O2 support after birth for at least 12 h; ^b^ wheeze, asthma diagnosis, or relevant medication up to two years before the enrollment; ^c^ systematic participation in sports.

**Table 2:** Spirometric characteristics.

	Term	Preterm (Combined)	Late Preterm (GA 34^0/7^–36^6/7^ Weeks)	Preterm (GA 29^0/7^–33^6/7^ Weeks)
FEV1, % pred.	97.8 ± 8.2 (82–114)	95.9 ± 9.3 (80–115)	96.6 ± 9.6 (80–115)	95 ± 9.2 (80–109)
FVC, % pred.	94.4 ± 11.1 (80–108)	93.2 ± 10.8 (73–108)	94 ± 10.5 (73–108)	92.3 ± 11.1 (73–101)
FEV1/FVC, %	0.92 ± 0.04 (0.83–0.99)	0.92 ± 0.04 (0.82–0.99)	0.92 ± 0.04 (0.82–0.99)	0.91 ± 0.03 (0.82–0.99)
FEF_25–75_, % pred.	96.6 ± 12.5 (77–132)	92.8 ± 11.4 (69–123)	94 ± 12.1 (73–123)	91.4 ± 10.2 (69–110)

Data presented as mean ± SD (range). There were no statistically significant differences between groups. FEV1: forced expiratory volume at 1 s, FVC: forced vital capacity, FEF_25–75_: forced expiratory flow at 25–75% of FVC, GA: gestational age.

**Table 3:** Cardiopulmonary parameters before CPET *.

	Term	Preterm (Combined)	Late Preterm (GA 340/7–366/7 Weeks)	Preterm (GA 290/7–336/7 Weeks)
RR (breaths per min)	20.5 ± 3.7 (14–29)	21.4 ± 4.1 (14–31)	21.4 ± 3.9 (15–31)	21.3 ± 4.5 (14–30)
VT (L)	0.53 ± 0.05 (0.44–0.61)	0.52 ± 0.06 (0.39–0.62)	0.53 ± 0.05 (0.43–0.61)	0.51 ± 0.06 (0.39–0.62)
VE (L/min)	11.4 ± 2.3 (7.5–14.8)	11.2 ± 2.2 (7.8–15.4)	11.5 ± 2.3 (7.8–15.4)	10.9 ± 2.1 (7.9–14.7)
HR (beats per min)	84.9 ± 9.3 (71–102)	84.9 ± 9.3 (71–102)	85.2 ± 9.2 (74–103)	87.5 ± 8.9 (75–105)
SpO_2_ (%)	99.3 ± 0.7 (98–100)	99.1 ± 0.8 (97–100)	99.3 ± 0.8 (97–100)	98.9 ± 0.8 (98–100)
RER	0.81 ± 0.05 (0.73–0.9)	0.82 ± 0.04 (0.75–0.91)	0.82 ± 0.05 (0.75–0.9)	0.83 ± 0.04 (0.77–0.91)

* Recorded during the accommodation phase before free pedaling. Data presented as mean ± SD (range). There were no statistically significant differences between groups. RR: respiratory rate, VT: tidal volume, VE: minute ventilation, HR: heart rate, SpO_2_: arterial blood saturation by pulse oximetry, RER: respiratory exchange ratio, GA: gestational age.

**Table 4:** CPET parameters.

	Term	Preterm (Combined)	Late Preterm (GA 34^0/7^–36^6/7^ Weeks)	Preterm (GA 29^0/7^–33^6/7^ Weeks)
Wmax, % pred.	88.6 ± 9 (72–105) ^a,b^	80.8 ± 8.9 (66–106) ^a^	83.7 ± 9.3 (70–106) ^b^	77.4 ± 7.1 (66–92) ^b^
HRmax, % pred.	92.1 ± 2.8 (87–103)	92.2 ± 3.3 (86–100)	92.1 ± 3.3 (86–100)	92.4 ± 3.4 (87–99)
VO_2_peak, % pred.	93.5 ± 16.3 (64–126) ^c,d^	84.6 ± 13.3 (60–118) ^c^	87.8 ± 13.9 (65–118) ^d^	80.9 ± 11.7 (60–104) ^d^
VO_2_ AT, % pred.	92 ± 13.7 (58–123)	89 ± 13 (59–112)	89.7 ± 13.6 (59–112)	88.2 ± 12.4 (60–109)
VO_2_/W, mL/min/Watt	12.5 ± 0.8 (10.9–14.1) ^e,d^	12.1 ± 0.8 (10.4–14.2) ^e^	12.3 ± 0.7 (11.1–14.2) ^d^	11.8 ± 0.7 (10.4–12.9) ^d^
O_2_ pulse, % pred.	100 ± 6.6 (86–114)	99.1 ± 7.1 (79–113)	99 ± 5.8 (88–111)	99.2 ± 8.5 (79–113)
RRmax, % pred.	81 ± 4.7 (73–92) ^f,g^	84.3 ± 5.6 (74–100) ^f^	83.3 ± 4.1 (74–93) ^g^	85.5 ± 6.9 (74–100) ^g^
VTmax, % pred.	80.2 ± 6.8 (68–95) ^h,i^	77.7 ± 5.7 (66–93) ^h^	78.7 ± 6.7 (68–93) ^i^	76.4 ± 4 (66–85) ^i^
VEmax, % pred.	80.5 ± 5.8 (68–93)	80.7 ± 5.2 (68–94)	80.2 ± 4.9 (71–94)	81.1 ± 5.7 (68–93)
Breathing reserve, %	29.9 ± 10.7 (9–53)	28.8 ± 9.7 (5–50)	28.9 ± 10.3 (5–50)	28.7 ± 9.1 (10–45)
VE/VCO_2_ slope, % pred.	99.4 ± 10.4 (67.4–126.2)	101.3 ± 11.9 (77–126.8)	100.4 ± 12.3 (77–126.8)	102.3 ± 11.6 (80–124.3)
OUES	1.95 ± 0.56 (0.95–3.2) ^j^	1.82 ± 0.54 (0.83–3.03)	1.89 ± 0.56 (0.95–3.03) ^j^	1.63 ± 0.42 (0.83–2.33) ^j^
SpO_2_max	98.4 ± 0.9 (97–100)	98 ± 1 (96–100)	98 ± 0.9 (97–100)	97.9 ± 1.2 (96–100)
PETCO_2_max (mmHg)	36.1 ± 2.7 (31–41)	35.9 ± 3.1 (30–42)	35.9 ± 3 (30–42)	35.8 ± 2.6 (30–41)
RER	1.16 ± 0.06 (1.06–1.26)	1.15 ± 0.05 (1.06–1.22)	1.15 ± 0.05 (1.06–1.23)	1.15 ± 0.06 (1.05–1.22)
Borg scale	7.3 ± 0.5 (6–9)	7.2 ± 0.5 (6–9)	7.2 ± 0.5 (6–9)	7 ± 0.4 (6–8)

Data presented as mean ± SD (range). *p*-value for comparisons between term and combined preterm (Mann–Whitney U test): ^a^ <0.001; ^c^ 0.003; ^e^ 0.003; ^f^ 0.001; ^h^ 0.017. *p*-value for comparisons between term, late preterm, and preterm (Kruskal–Wallis test): ^b^ 0.002; ^d^ 0.002; ^g^ 0.004; ^i^ 0.025; ^j^ 0.038. W: work, HR: heart rate, VO_2_: O_2_ consumption, AT: anaerobic threshold, RR: respiratory rate, VT: tidal volume, VE: minute ventilation, OUES: O_2_ uptake efficiency slope, SpO_2_: arterial blood saturation by pulse oximetry, PETCO_2_: end-tidal CO_2_ tension, RER: respiratory exchange ratio, GA: gestational age.

## Data Availability

The data presented in this study are available on reasonable request from the corresponding author. The data are not publicly available due to restrictions imposed by the Ethics Committee of the University Hospital of Patras.

## References

[1] Cao G., Liu J., Liu M. (2022). Global, regional, and national incidence and mortality of neonatal preterm birth, 1990–2019. JAMA Pediatr..

[2] Pravia C.I., Benny M. (2020). Long-term consequences of prematurity. Clevel. Clin. J. Med..

[3] Ashorn P., Ashorn U., Muthiani Y., Aboubaker S., Askari S., Bahl R., Black R.E., Dalmiya N., Duggan C.P., Hofmeyr G.J. (2023). Small vulnerable newborns-big potential for impact. Lancet.

[4] Simpson S.J., Logie K.M., O’Dea C.A., Banton G.L., Murray C., Wilson A.C., Pillow J.J., Hall G.L. (2017). Altered lung structure and function in mid-childhood survivors of very preterm birth. Thorax.

[5] Welsh L., Kirkby J., Lum S., Odendaal D., Marlow N., Derrick G., Stocks J., EPICure Study Group (2010). The EPICure study: Maximal exercise and physical activity in school children born extremely preterm. Thorax.

[6] Kaplan E., Bar-Yishay E., Prais D., Klinger G., Mei-Zahav M., Mussaffi H., Steuer G., Hananya S., Matyashuk Y., Gabarra N. (2012). Encouraging pulmonary outcome for surviving, neurologically intact, extremely premature infants in the postsurfactant era. Chest.

[7] Rideau Batista Novais A., Matecki S., Jaussent A., Picot M.C., Amedro P., Guillaumont S., Picaud J.C., Cambonie G. (2012). Hyperventilation during exercise in very low birth weight school-age children may implicate inspiratory muscle weakness. J. Pediatr..

[8] Clemm H., Røksund O., Thorsen E., Eide G.E., Markestad T., Halvorsen T. (2012). Aerobic capacity and exercise performance in young people born extremely preterm. Pediatrics.

[9] MacLean J.E., DeHaan K., Fuhr D., Hariharan S., Kamstra B., Hendson L., Adatia I., Majaesic C., Lovering A.T., Thompson R.B. (2016). Altered breathing mechanics and ventilatory response during exercise in children born extremely preterm. Thorax.

[10] Kriemler S., Keller H., Saigal S., Bar-Or O. (2005). Aerobic and lung performance in premature children with and without chronic lung disease of prematurity. Clin. J. Sport Med..

[11] Pianosi P.T., Fisk M. (2000). Cardiopulmonary exercise performance in prematurely born children. Pediatr. Res..

[12] Joshi S., Powell T., Watkins W.J., Drayton M., Williams E.M., Kotecha S. (2013). Exercise-induced bronchoconstriction in school-aged children who had chronic lung disease in infancy. J. Pediatr..

[13] O’Dea C.A., Logie K., Maiorana A., Wilson A.C., Pillow J.J., Banton G.L., Simpson S.J., Hall G.L. (2018). Increased prevalence of expiratory flow limitation during exercise in children with bronchopulmonary dysplasia. ERJ Open Res..

[14] Ruf K., Thomas W., Brunner M., Speer C.P., Hebestreit H. (2019). Diverging effects of premature birth and bronchopulmonary dysplasia on exercise capacity and physical activity—A case control study. Respir. Res..

[15] Edwards M.O., Kotecha S.J., Lowe J., Watkins W.J., Henderson A.J., Kotecha S. (2015). Effect of preterm birth on exercise capacity: A systematic review and meta-analysis. Pediatr. Pulmonol..

[16] Hochwald O., Bentur L., Haddad Y., Hanna M., Zucker-Toledano M., Mainzer G., Haddad J., Gur M., Borenstein-Levin L., Kugelman A. (2022). Cardiopulmonary exercise testing in childhood in late preterms: Comparison to early preterms and term-born controls. J. Pers. Med..

[17] Weigelt A., Bleck S., Huebner M.J., Rottermann K., Waellisch W., Morhart P., Abu-Tair T., Dittrich S., Schoeffl I. (2023). Impact of premature birth on cardiopulmonary function in later life. Eur. J. Pediatr..

[18] Vrijlandt E.J., Gerritsen J., Boezen H.M., Grevink R.G., Duiverman E.J. (2006). Lung function and exercise capacity in young adults born prematurely. Am. J. Respir. Crit. Care Med..

[19] Chawanpaiboon S., Vogel J.P., Moller A.B., Lumbiganon P., Petzold M., Hogan D., Landoulsi S., Jampathong N., Kongwattanakul K., Laopaiboon M. (2019). Global, regional, and national estimates of levels of preterm birth in 2014: A systematic review and modeling analysis. Lancet Glob. Health.

[20] Osterman M.J.K., Hamilton B.E., Martin J.A., Driscoll A.K., Valenzuela C.P. (2023). Births: Final Data for 2021.

[21] NHS (2019). Physical Activity Guidelines for Children and Young People. https://www.nhs.uk/live-well/exercise/physical-activity-guidelines-children-and-young-people/.

[22] Cole T.J., Lobstein T. (2012). Extended international (IOTF) body mass index cut-offs for thinness, overweight and obesity. Pediatr. Obes..

[23] Papathanasiou G., Georgoudis G., Papandreou M., Spyropoulos P., Georgakopoulos D., Kalfakakou V., Evangelou A. (2009). Reliability measures of the short International Physical Activity Questionnaire (IPAQ) in Greek young adults. Hell. J. Cardiol..

[24] Miller M.R., Hankinson J., Brusasco V., Burgos F., Casaburi R., Coates A., Crapo R., Enright P., van der Grinten C.P., Gustafsson P. (2005). Standardisation of spirometry. Eur. Respir. J..

[25] Quanjer P.H., Stanojevic S., Cole T.J., Baur X., Hall G.L., Culver B.H. (2012). ERS Global Lung Function Initiative. Multi-ethnic reference values for spirometry for the 3–95-yr age range: The global lung function 2012 equations. Eur. Respir. J..

[26] Godfrey S., Davies C.T., Wozniak E., Barnes C.A. (1971). Cardio-respiratory response to exercise in normal children. Clin. Sci..

[27] Paridon S.M., Alpert B.S., Boas S.R., Cabrera M.E., Caldarera L.L., Daniels S.R., Kimball T.R., Knilans T.K., Nixon P.A., Rhodes J. (2006). Clinical stress testing in the pediatric age group: A statement from the American Heart Association Council on Cardiovascular Disease in the Young, Committee on Atherosclerosis, Hypertension, and Obesity in Youth. Circulation.

[28] Pianosi P.T., Smith J.R. (2019). Ventilatory limitation of exercise in pediatric subjects evaluated for exertional dyspnea. Front. Physiol..

[29] Burstein D.S., McBride M.G., Min J., Paridon A.A., Perelman S., Huffman E.M., O’Malley S., Del Grosso J., Groepenhoff H., Paridon S.M. (2021). Normative values for cardiopulmonary exercise stress testing using ramp cycle ergometry in children and adolescents. J. Pediatr..

[30] American Thoracic Society, American College of Chest Physicians (2003). ATS/ACCP Statement on cardiopulmonary exercise testing. Am. J. Respir. Crit. Care Med..

[31] Lagiou O., Fouzas S., Lykouras D., Sinopidis X., Karatza A., Karkoulias K., Dimitriou G., Anthracopoulos M.B. (2022). Exercise limitation in children and adolescents with mild-to-moderate asthma. J. Asthma Allergy.

[32] Dempsey J.A., Romer L., Rodman J., Miller J., Smith C. (2006). Consequences of exercise-induced respiratory muscle work. Respir. Physiol. Neurobiol..

[33] Melamed K.H., Santos M., Oliveira R.K.F., Urbina M.F., Felsenstein D., Opotowsky A.R., Waxman A.B., Systrom D.M. (2019). Unexplained exertional intolerance associated with impaired systemic oxygen extraction. Eur. J. Appl. Physiol..

[34] Baba R., Nagashima M., Goto M., Nagano Y., Yokota M., Tauchi N., Nishibata K. (1996). Oxygen uptake efficiency slope: A new index of cardiorespiratory functional reserve derived from the relation between oxygen uptake and minute ventilation during incremental exercise. J. Am. Coll. Cardiol..

[35] Wasserman K. (1986). The anaerobic threshold: Definition, physiological significance and identification. Adv. Cardiol..

